# The Sensory Space of Wines: From Concept to Evaluation and Description. A Review

**DOI:** 10.3390/foods10061424

**Published:** 2021-06-19

**Authors:** Jean-Christophe Barbe, Justine Garbay, Sophie Tempère

**Affiliations:** Unité de Recherche Œnologie, ISVV, Université de Bordeaux, EA 4577, USC 1366 INRAE, F33882 Villenave d’Ornon, France; justine.garbay@u-bordeaux.fr (J.G.); sophie.tempere@u-bordeaux.fr (S.T.)

**Keywords:** sensory characterization, sensory space, descriptive sensory analysis, concept evaluation, wine

## Abstract

The concept of sensory space was first formulated over 25 years ago and has been widely adopted in oenology for around the last 15 years. It is based on both the common organoleptic characteristics of products and the mental representations built by specific groups of people. Exploring this concept involves first assessing whether it already exists for tasters, and, when this is the case, conducting perceptual evaluations to verify its effectiveness before potentially highlighting the associated sensory properties. The goal of this review, which focuses on applications linked to the field of oenology, is to study how these three steps are carried out, how the corresponding tasks and tests are performed and managed, and the type of results that can be obtained.

## 1. Introduction

From the beginning of the 19th century to the mid-20th century, wine tasting was epitomized by a poor vocabulary that mainly focused on the visual aspect and mouthfeel. In 1952, Maynard A. Amerine introduced the aromatic evaluation of wine with his Californian wine tasting card. Sensory evaluation was developed in France between the 1950s and the 1970s as a tool to validate protected designated origin wines, resulting in a need for words to describe typicality. The first approaches involved the development of descriptive analyses that generally used natural products as references [1].

The notion of sensory space was introduced later by Dacremont and Vickers [2] and was defined for wine by Candelon et al. [3] as the common character of different aromatic expressions that would subsist beyond all diversity. According to Picard [4], a sensory space is a relatively broad notion based on sensory criteria and mental representations of a product constructed by a group of individuals. Many studies have adopted the characterization of sensory space in order to explore sensory concepts. For instance, wine typicality or complexity, as well as aging potential, are considered as sensory concepts, and have been the focus of numerous oenological studies [5,6,7,8].

Moreover, investigations on sensory space can also demonstrate the influence (or lack thereof) of the tasters’ expertise on the perceived sensory characteristics of products, helping to characterize the tasting learning in oenology [9,10].

**Figure 1:**
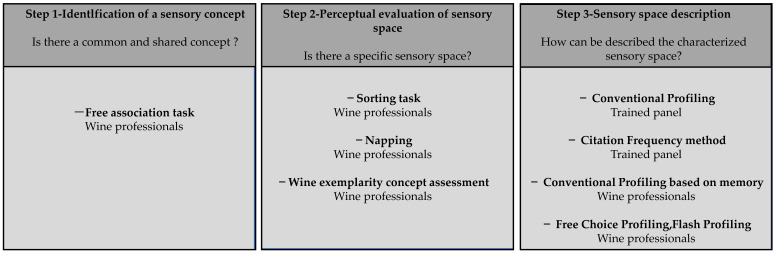
Three-step approach for sensory space characterization: from concept to evaluation and description.

The aim of this paper is to gather, explain, and discuss methods that allow us to define and describe a sensory space. To this end, various conceptual and perceptual approaches will be explored, along with illustrations of their use in oenology. The characterization of a sensory space can be divided into three phases (Figure 1). The first step involves a conceptualization task in order to evaluate whether a sensory concept exists (i.e., if it is shared by different subjects). If the sensory concept is established, the second step involves a perceptual approach dedicated to objectively assessing the effectiveness of the corresponding sensory space. Finally, if this space is defined, the third step comprises a sensory approach in order to describe the associated sensory properties.

The present review has been organized according to this three-step approach (Figure 1). In the first part, conceptual methods such as free association tasks used for the identification of a sensory concept will be considered. The second and the third parts respectively present the evaluation and description of a sensory space according to perceptual approaches. Sorting tasks based on similarities between samples are mainly used to assess the existence of a sensory space, whereas descriptive methods, such as conventional profiles or free choice profiling, are generally performed to characterize it.

## 2. Identification of a Sensory Concept (Step 1)

The notion of sensory concept can be defined as the mental representation of the characteristics of a food product category [9]. In order to apply sensory concepts to specific products, it is important to understand how they can be imagined and defined by individuals. The existence of a sensory concept can therefore only be demonstrated through conceptual approaches that do not require the use of either sensory perception or sensory samples.

A free association task, also known as word association, is one of the most common qualitative methods used to identify individual mental representations and to study the conceptual dimensions of objects or sensory concepts [11]. 

During an interview performed by the experimenter, participants write words or expressions on a sheet of paper to spontaneously express what comes to mind when asked to describe a specific subject [12]. In most cases, the instructions are presented both orally and in written form. For example, Langlois et al. [6] applied a two-question approach, which led to both an association task and a definition task: “What comes to mind when I say “wine with aging potential”?”, “What do you associate with “wine with aging potential”?”, and “If you have to give a definition of “wine with aging potential”, for you, a “wine with aging potential” would be...” The directives for the association task could also be in the form of a role-play, as proposed by Ginon et al. [13]: “Imagine that you are in your usual retail store and about to buy a bottle of wine. Please list all the criteria you generally take into account in this situation. You can cite as many expressions or terms that come spontaneously to mind”, or as Picard et al. [8] suggested: “Imagine you have to explain to a friend what a reductive ageing bouquet in red Bordeaux wines is. What would be the most suitable descriptors to define it sensorially? A limit of 5 descriptors is required.” 

This conceptual approach can be combined with variants in order to complete the evaluation of an object or a specific concept [7]. Generally, the next step is an associated verbalization task, which consists of a free description of the lexical field generated during word association. Participants then have to assess the importance of words through a ranking task in accordance with the instructions proposed by Parr et al.: “Now, please prioritize the words you have just given in order of importance, with 1 being the highest in importance.” Finally, a valence rating (an evaluation of the correlation between the words and the concept) is performed on a seven-point scale, anchored from the left end −3 (extremely negative) to the right end +3 (extremely positive).

Data obtained from a free association task may be analyzed by a two-step approach. Generally, words or expressions written by each participant are not directly usable and have, first, to be transformed according to semantic analysis as described by Symoneaux et al. [14]. This procedure mainly consists of removing spelling mistakes, cancelling connectors and auxiliary terms, and categorizing synonyms or words linked to the same lexical field. Hough and Ferraris [12] recommended eliminating words and sentences cited by just a few participants as these words were not relevant. This semantic analysis is generally performed independently by wine researchers according to their own categorization criteria, and categories and labels for each category are established by consensus. Finally, citation frequency is determined by two parameters according to the following formula proposed by the occurrence frequency OF = ON/N, where ON is the number of times a word is cited and N the total number of words cited, while the percentage of participants who cite the same word is PF = FN/N, where FN is the number of participants who cite the same word in question, and N the total number of participants [8]. Citation frequency is evaluated in order to identify the most relevant words or expressions used by participants. Textual data analysis software, such as ALCESTE (I.M.A.G.E., Toulouse, France), may serve as an alternative to realize this semantic analysis [15]. To take the interpretation of the results further, statistical analyses of semantic data may be performed, starting with the construction of a distance matrix in order to apply multidimensional scaling (MDS) and hierarchical cluster analysis (HCA) [12].

A few oenological studies have used free association tasks. These suggest that, depending on their goals, the selection of a suitable panel is important. Picard et al. [8] included a free association method in their sensory approach to explore the concept of the ageing bouquet in red Bordeaux wines via a specific panel. The panel was made up of experts who shared a sensory definition of the aging bouquet, structured around eight main aromatic notes.

Parr et al. [7] used a free association task to investigate the influence of expertise on the mental representations of complexity in wine. The aim was to see whether an expert’s mental representation of a specific sensory concept was similar to a novice’s mental representation of the same sensory concept. The panels of novices or consumers differ from experts as they encompass people who drink wine between once a day and once a month and have no experience in wine evaluation or winemaking. The main criteria highlighted by novices to characterize complexity in wine were linked to the quality (olfactory and gustatory properties) and the brand of the wine. Conversely, wine professionals associated the concept of wine complexity to factors dependent on vine, soil, and winemaking. Mental representations of complexity in wine were thus shown to be different between experts and novices. In similar vein, Rodrigues et al. [16] performed a comparative study of the mental representations of wine minerality between wine consumers and wine professionals. They argued that mental representations of minerality by experts and novices were similar since the most important criteria proposed by both panels to characterize this concept were related to the geological composition of soils and the sensory properties of the wines. 

## 3. Perceptual Evaluation of Sensory Space (Step 2)

Once the existence of a sensory concept has been demonstrated, it can be evaluated and confirmed from a perceptual point of view. Such approaches allow us to understand and highlight sensory boundaries among samples. Sensory boundaries can be defined as categories that include samples which share many similarities and many dissimilarities with samples from other categories [9]. Samples with sensory similarities belonging to the same category may be defined as being sensory spaces. 

Many perceptual approaches allow us to evaluate perceptive similarities and differences between samples in order to create sensory borders and thus define sensory spaces. They are summarized in Table 1. Such sensory techniques are generally combined with verbalization tasks in order to understand the participants’ categorization strategy [17].

In this part of the process, all the experts on the panel are professionals according to the criteria proposed by Parr et al. [18,19], with no specific training.

### 3.1. Sorting Tasks

A sorting task is a qualitative method that consists of grouping samples based on their similarities or dissimilarities. This sensory approach is a fast and efficient method to obtain information about perceptual differences between samples belonging to a specific product [20]. For Cartier et al. [21], a sorting task is mainly used in the sensory assessment of products as it is less time-consuming than other methods and reduces fatigue or boredom for the assessors. In addition, according to Piombino et al. [22], one of the main advantages of this method is the possibility of evaluating a large number of samples in just one session. The first use of a sorting task was the evaluation of perceptual odor quality [23]. Lelièvre et al. [24] explained that a sorting task is based on a natural and regular cognitive process called categorization. By identifying sensory similarities and dissimilarities between samples belonging to a sensory concept, it is possible to study a sensory space. For Bécue-Bertaut et al. [25], a sorting task provides an assessment of a sensory product from a global perspective and allows for comparisons between different participant behaviors (experts or consumers, for example). 

Several versions of sorting tasks have been developed in the sensory field, but there are two main variations in oenological applications: the free sorting task and the directed sorting task [26]. Sorting tasks may be used alone or in combination with a verbalization task where participants must define each group and how these are formed [27]. 

#### 3.1.1. Free Sorting Tasks

Samples are randomly presented to the participants and the assessors must first smell and taste each sample once in the proposed order. They are then allowed to smell and taste samples as many times they wish and in any order. There are no constraints in the free sorting task. For example, Bécue-Bertaut et al. [25] suggested using a sorting task to compare the categorization strategy of red Catalan wines through a cross-cultural study, focusing on categorization by French and Spanish wine professionals. For example, the experimenter gave the assessors the following instruction: “Eight red Catalan wines are placed in front of you. You are asked to group them into clusters of wines that you consider alike, depending on your own criteria. You have to compose at least two clusters and no more than seven. Then, you can associate the words that better characterize them to every wine or cluster.”

Analyzing data obtained by each participant after a free sorting task allowed for the construction of an individual matrix of similarities where rows and columns corresponded to samples. When a participant had grouped two samples together, a value of 1 was attributed at the intersection of a row and a column on the matrix. However, when two samples were not gathered, a value of 0 was attributed at the intersection of a row and a column. Then, individual matrices were added in order to obtain a global matrix of similarities. Finally, the global matrix was analyzed by MDS and HCA in order to visualize how the sorting task was performed by panelists.

Many oenological studies have used free sorting tasks to explore perceptive similarities between samples related to a sensory concept, and to define a specific sensory space [28]. Ballester et al. [5] investigated the concept of typicality, asking novices (students from the University of Bourgogne) to categorize 18 wines related to the Chardonnay wine concept. The authors decided to present nine wines with a low degree of typicality of the Chardonnay wine concept and nine wines with a high degree of typicality. The results of the ortho-nasal assessment failed to determine a specific category of the Chardonnay wine concept, apart from a Sauvignon wine which was located very far from others on the map obtained by MDS. To continue investigating the concept of typicality through perceived odors and to understand the influence of expertise on categorization, Ballester et al. [9] asked experts and novices to compare the way they sorted Melon de Bourgogne wines and Chardonnay wines. The study showed that experts clearly distinguished Melon de Bourgogne from Chardonnay wines following an ortho-nasal assessment, while novices could not clearly separate them. The authors concluded that the experts used a top-down strategy based on their wine knowledge, while novices sorted samples only through perceptual characteristics as they did not have the wine expertise required. Experts had a common mental representation of both types of wine, while novices had no common representation.

Ballester et al. [29] tried to determine whether a trained panel could properly categorize wine samples according to their color (white, red, or rosé wines) in the dark through a free sorting task. Training sessions lasted from 8 months to 2 years and included work on the memorization of common olfactory references. The results showed that trained participants clearly distinguished white wines from red wines, but not rosé wines. This confused sorting outcome may be explained by the fact that rosé and white wines present olfactory similarities, and that both red and rosé wines come from red grape varieties.

Working with a trained panel, Campo et al. [30] tried firstly to define the sensory space of young Spanish monovarietal white wines through a free sorting task based on olfactory similarities, and secondly to understand the influence of grape varieties on the aroma of young Spanish monovarietal wines. Training sessions on the identification of common odor were performed in two 1 h sessions a week. The results showed that there were no clear categories based on grape variety and the authors concluded that grape varieties were not the main factor affecting the sensory space of young Spanish monovarietal white wines. 

#### 3.1.2. Directed Sorting Tasks

A directed sorting task consists of providing criteria or instructions on the number and/or the nature of groups. Chollet et al. [26] described the directed sorting task as a variation of the sorting task, whereby participants provide a discriminatory assessment between different categories of a specific product. The tasters may be offered a list of descriptors to help them to sort the wines, for example. After sorting, the panelists are asked to select the most relevant terms from the list to define each group [31]. 

Binary sorting tasks are mainly used to define the membership of a sample to a specific sensory space by categorizing samples into two groups with a focus on their dissimilarities, as described by Cartier et al. [21]. Constraints applied to assessors in a binary sorting task depend on the experimenter’s expectations. The ternary sorting task is an extension of the binary sorting task using a very similar method, but in this case, assessors have to form three groups instead of two. 

To analyze binary sorting data, an individual dissimilarity matrix is first constructed, and each individual matrix is then added. These individual matrices allow identification when two samples are grouped together. Thus, a grouping matrix is submitted to MDS. Groups are then identified using HCA [32].

A few studies have used a binary ortho-nasal sorting task to investigate wine typicality concepts. For example, Parr et al. [31] explored the sensory characteristics of New Zealand Sauvignon Blanc wines. The panel, composed of wine professionals selected for their interest and their high level of expertise with regard to Sauvignon Blanc wines, had to sort samples into “good varietal definition” or “not good varietal definition”. The study demonstrated that wines categorized as “good varietal definition” were considered to express Sauvignon Blanc wine typicality. Ballester et al. [29] adopted a ternary ortho-nasal sorting task to identify the influence of level of expertise on the categorization of red, white and rosé wines. The assessors were instructed to sort each wine sample by smelling them in the order they were presented. Thus, and as previously noted (cf. Section 3.1.1. Free sorting tasks), experts, novices, and trained panelists were able to characterize red and white wines well, while the categorization of rosé wines was more challenging. The concept of the aging potential of red Burgundy wines was also explored through binary sorting tasks by Jaffré et al. [32] in order to define its perception and representation by wine professionals. The experimenter gave a simple instruction: the participants had to sort samples according to whether they had the potential to age or not. The assessors were allowed to smell and taste the samples. The authors showed that experts categorize wines using visual and olfactory-gustatory assessments: wines presenting astringency and an intense color were generally sorted as having a potential for ageing, while wines with less astringency and a light color were categorized as without the potential to age. Taking this further, Langlois [33] suggested the use of binary sorting tasks in two experimental conditions: (1) an ortho-nasal evaluation and (2) a global evaluation with visual, olfactory, and gustatory assessments. The results were similar to those obtained by Jaffré et al. [32]. 

### 3.2. Projective Mapping or Napping

Projective mapping is a rapid and qualitative sensory approach introduced in the sensory field by Risvik et al. [34], and provides a two-dimensional perceptual map. Participants have to place samples on a given space (generally an A4 or A3 sheet of paper) according to the sensory similarities and dissimilarities they perceive. The closer the samples are placed, the more they are deemed to be similar. If samples are perceived as different, they are placed far apart on the sheet. The samples are placed next to each other, and the assessors discriminate the samples according to their own criteria. 

Napping is a specific form of projective mapping introduced by Pagès [35]. Specifically, the sample space is a rectangular paper tablecloth (40 cm × 60 cm, globally A2 size). The term “Napping” comes from the word “nappe”, which means “tablecloth” in French [36]. Dehlholm et al. [37] proposed two approaches to Napping: global Napping and partial Napping. Global Napping involves the global assessment of samples, while partial Napping is performed with regard to just one sensory criterion, such as visual aspect, smell, or tasting evaluation. According to Perrin et al. [38], Napping can be considered as a sorting task and is useful in a sensory concept assessment. This sensory approach provides a global sensory image of a product and can give an overall assessment of a sensory concept. Pagès offers an example of the instructions given to participants [36]: “You are asked to evaluate the similarities (or dissimilarities) between several wines. You have to do this according to your own criteria, those that are significant for you. You do not have to indicate your criteria. There is no good or bad answer. You have to position the wines on the tablecloth in such a way that two wines are very near if they seem identical to you and two wines are distant from one another if they seem different to you. This must be done according to your own criteria. Do not hesitate to express strongly the differences you perceive by using most of the sheet. When the operation is finished, write on the sheet the number of the wine in the place it occupies.”

Napping data are obtained by collecting X and Y coordinates for each sample. Generally, the ordinate chosen is the left bottom corner of the tablecloth. The table of coordinates gathers all the participants’ tablecloths. Concerning the statistical analysis of the data, Pagès [36] suggested that several statistical analyses could be performed. However, the main form of statistical analysis used for Napping data is multiple factor analysis (MFA).

Pagès [36] used a global Napping approach to explore white wines from the Loire Valley. Samples were submitted to wine professionals (winegrowers, oenologists, and wine waiters). The study demonstrated how the two-dimensional perception differed from one participant to another, information that appeared difficult to obtain in another way. The Napping results revealed that there was no overall agreement between assessors and suggested that sensory representations of wine samples from the Loire Valley are categorized differently between experts.

### 3.3. Wine Exemplarity Concept Assessments

This approach has been used in particular to study the notion of typicality. The experimenter gave a specific instruction to participants based on the following example [3]: “Imagine that you must explain to a friend what is a wine produced from Sciaccarello. To explain, you can have him taste a wine. For each wine presented, you must answer the following question: Do you consider that this wine is a good or a bad example to illustrate to your friend what is a wine produced from Sciaccarello?” In the instructions, the experimenter added that there were no right or wrong answers: “For different reasons due to growing or oenological practices, it is possible that Sciaccarello wine does not seem to you a good example of what can be obtained from this variety. On the other hand, wine made with another variety could seem to you a good example. Our interest is in your personal appreciation.” The participants were required to answer on an unstructured scale, graduated from the left-end “very bad example” to the right-end “very good example”, where good examples were perceived as the most typical of the concept under consideration. 

The data obtained from the scales were converted to scores. Generally, 10 cm scales were used and scores of 0 to 10 were obtained [5]. Principal component analysis (PCA) was performed in order to determine a consensus among the assessors, and HCA meant that the distribution of samples could be observed on a map along a horizontal axis. Finally, analysis of variance (ANOVA) was conducted to classify wines into the “good example” or “bad example” categories and to determine if any significant differences between samples could be highlighted. PCA also allowed for an evaluation of the level of consensus between tasters [9].

Ballester et al. [5] used the same process to investigate the Chardonnay wine concept with a panel of experts. They showed that it was characterized by a specific aroma and mouthfeel perceptions. Similarly, the typicality of New Zealand Sauvignon Blanc was assessed by Parr et al. [31,39]. First, the authors investigated the distinctive “Malborough Sauvignon Blanc” style by ortho-nasal and global evaluations of Sauvignon Blanc wines from New Zealand and France. The panel was composed of New Zealand experts selected on the basis of having a high experience in Sauvignon Blanc wines. Parr et al. [39] performed the same study but with a panel of French experts. The results were similar for both panels, and a sensory border between New Zealand and French Sauvignon Blanc wines was determined. Sarrazin [40] explored the sensory properties of sweet white wines from Bordeaux by comparing such wines from Bordeaux with sweet white wines from other areas and dry white wines from Bordeaux. The approach led to the determination of three distinct categories representing the three types of wines studied and proved the existence of a sensory space that characterized the typicality of Bordeaux sweet white wines. Pineau et al. [41] investigated the fruity expression of Bordeaux red wines from cv Merlot Noir and Cabernet Sauvignon. Several wine samples from different origins were presented to the assessors: red wines from Bordeaux, red wines from other areas, and white wines. The results highlighted the existence of a Bordeaux typicality as Bordeaux red wines were gathered into one group, whereas other samples were separated into other groups. A similar approach was used for other sensory concepts such as the aging bouquet of red Bordeaux wines [8].

**Table 1:** Inventory of perceptual methodologies used to evaluate sensory space. Statistical analysis—MDS: multi-dimensional scaling; HCA: hierarchical cluster analysis; MFA: multiple factor analysis; PCA: principal component analysis; ANOVA: analysis of variance.

Sensory Test	Data Collected	Statistical Analysis	Advantages	Disadvantages
**Sorting task [20,27]**	Similarity or dissimilarity matrix	❖ MDS ❖ HCA	❖ Less time-consuming [21]. ❖ Limits fatigue [21]. ❖ Evaluates a larger number of samples in 1 session [22]. ❖ No specific training for participants. ❖ Possibility of combination with a verbalization task.	❖ Only provides a comparative presentation of samples. ❖ Is an intuitive technique for panelists but there are possible difficulties with regard to memorization and criteria choice in the formation of groups.
**Projective mapping or Napping [34]**	Coordinates table of each sample	❖ MFA [36]	❖ Less time-consuming ❖ No specific training for participants. ❖ Possibility of combination with ultra-flash profiling.	❖ *cf. Sorting task*
**Wine exemplarity concept assessment [3]**	Intensity rating	❖ PCA [9] ❖ HCA ❖ ANOVA	❖ Global approach related to the classic tasting carried out by professionals	❖ The need to apply this method with professionals.

## 4. Sensory Space Description (Step 3)

Descriptive sensory analyses or descriptive analyses encompass quantitative and qualitative approaches, and lead to a sensory description of products through an analytical judgement. Descriptive analyses are mainly applied in sensory profiles when a detailed description of a product’s sensory attributes is sought or when the sensory properties of different products have to be compared. However, depending on the descriptive techniques used, the sensory description may be more or less objective, and may be qualitative or quantitative [42].

Two main descriptive methodologies are generally considered according to the way data are gathered (generation of marks on a scale or determination of descriptor citation frequencies), but modified approaches have also been developed. All of them are summarized in Table 2.

### 4.1. Conventional Profiling Methods

Conventional profiling (CP), also called conventional descriptive analysis or quantitative descriptive analysis, is a classical method introduced by Stone et al. [43] to describe the sensory characteristics of products based on intensity ratings of descriptors. Sensory profiles are used to identify the main qualitative and quantitative sensory dimensions of products. In CP it is important to perform these approaches with an extensively trained panel in order to obtain consistent and reproducible results.

The CP process can be divided into three main steps. The first step is the extensive training of assessors proposed by Dairou and Sieffermann [44]. The training sessions begin by familiarizing the panel with the product’s sensory space, and a range of samples are presented to each participant individually. The assessors are asked to generate terms describing their perception and to classify them according to different modalities: visual aspect, smelling, and tasting assessments. The descriptors are pooled and discussed with the tasters and the experimenter in order to reach a consensus on which specific descriptors will then be used. Thus, the initial list is reduced in order to obtain an understandable and specific list of descriptors. References are defined for these descriptors following consensus of the panelists, who then train to use them, including use of the intensity rating scales. Pelonnier-Magimel et al. [45] suggested specific and adapted sensory training divided into five sessions, with each session lasting approximately one hour. Session 1 consists of validating references prepared from corresponding natural products which can be used to confirm the use of descriptors previously generated by the panel. Session 2 repeats session 1 and proposes a recognition phase where the assessors have to evaluate the references and associate them with previously generated descriptors. Session 3 suggests an intensity evaluation of the descriptors. In session 4, “product characterization training”, where the participants have to taste and evaluate wine samples, is recommended. Finally, in session 5, presented as “training to recognize references in wine”, the panel has to evaluate references and wines supplemented with these references or not. The second step of the CP process consists of monitoring the participants’ performance by determining their discriminating power, their reproducibility and their homogeneity. The third and final step consists of individual evaluations (orthonasal and/or global evaluations) of samples, including randomly presented sample replications. The experimenters use replications in order to assess whether the participants can discriminate samples or if they need more training. During these sample assessments, the assessors are asked to rate the intensity of each attribute on an unstructured or a structured scale.

Data analysis can be separated into individual and collective treatments [42]. The individual analysis is performed using ANOVA applied to descriptive ratings. Samples and replications are applied as sources of variation. ANOVA evaluates the reliability of individual participants with respect to a variant (sample or replication), determining whether a participant is consistent or not. For the collective treatment, ANOVA with interactions is also performed in order to identify interactions between participants and samples. For attributes that show a significant interaction effect between the assessor and the sample, PCA is applied to the correlation matrix in order to assess the disagreement between participants [43].

Many authors have used conventional profiling in the oenological field to describe wine sensory spaces, even if many of them omit or adapt the steps of descriptor generation and panel training. For instance, in their studies on the distinctive New Zealand “Marlborough Sauvignon Blanc” wine style, Parr et al. [31] determined a CP based on intensity by ortho-nasal and global evaluations of wine samples from New Zealand and France. The assessors had to rate the intensity of 10 previously selected flavor descriptors [46]. Thus, “Marlborough Sauvignon Blanc” wine typicality was characterized by passion fruit, boxwood, and green capsicum notes.

Using an exhaustive methodology, Pelonnier-Magimel et al. [45] compared wines from two maturity levels of Merlot N, produced with or without the addition of SO_2_. First, the results showed that wines produced at technological maturity were characterized by “red fruit notes”, whereas wines with advanced maturity presented “jammy fruit” notes and “astringency” characteristics. Wines made without the addition of SO_2_ were characterized by “freshness” (“mint” and “coolness” descriptors) and “cooked black cherries”. However, wines protected by SO_2_ were described as ‘smoky” [45]. 

Moreover, an approach derived from CP could also be conducted on memory alone (without wine tasting and based only on mental wine representations in memory). Jose-Coutinho et al. [47] suggested using this approach to characterize various Portuguese Protected Geographical Indications. In this case, “the descriptive analysis” was performed by wine professionals and was used as a means to approach a sensory concept.

### 4.2. Variations of Conventional Profiling

These methodologies were adapted from conventional profiles to provide easier solutions. They use, in particular, a panel of wine professional experts for which specific training is not required. More globally, the topic of easy and rapid profiling methods has been studied by Brand [48].

#### 4.2.1. Free Choice Profiling

Langron [49] introduced free choice profiling (FCP) as an alternative to conventional profiling (CP). This profiling technique provides a sensory description of samples based on the free generation of vocabulary, allowing a relative ranking of samples [50]. Participants are required to generate their own attributes that they consider important to describe the products properly and to avoid the omission of major sensory characteristics. FCP is seen as a simplified form of CP since this sensory descriptive method does not require training sessions. The process is very simple as panelists evaluate samples in one session orthonasally and/or globally, freely describing the sensory properties they perceive by creating their own list of attributes without the need to explain the significance of the words. Participants can use as many words or expressions as they want and receive no instructions on the strategy to describe samples, apart from avoiding hedonic attributes [49].

The list of descriptors obtained after FCP must be transformed according to the criteria of Symoneaux et al. [14] in order to correct assessors’ mistakes and to eliminate synonyms. Experimenters classify attributes into main modalities with the aim of facilitating data analysis and interpretation of the results. Descriptors are added to a table with rows corresponding to attributes, columns to wines and, at the intersection of rows and columns, the number of citations of each descriptor. Only terms cited by a minimum of 20% of participants are taken into account [51].

The statistical analysis of data begins with a global χ² test to assess the dissimilarities perceived by the participants. A correspondence analysis is then applied to the data recorded in a contingency table in order to visualize the interactions between the samples and descriptors. Generalized Procrustes analysis (GPA) can be performed to determine the terms used by individual panelists that appear to measure the same sensory attributes as the other participants. Moreover, through individual spatial representation, this form of statistical analysis helps to determine how the different terms used by different assessors may be interrelated [42].

Perrin et al. [50] suggested comparing CP and FCP using a panel of white Chenin Blanc wine experts. The principal advantages of FCP are its rapidity and flexibility, which allows it to be used with wine professional experts as the latter are rarely able to participate in extensive training due to their lack of time. FCP techniques do not require extensive training and allow experts to evaluate samples in one session. The results of this study proved that the key descriptors highlighted by CP were also found through FCP. Thus, this free descriptive method appears to be suitable for such a panel. Nevertheless, the authors admitted that CP is better to evaluate small sensory differences between samples, providing greater accuracy.

#### 4.2.2. Flash Profiling

Flash profiling (FP) was invented by Sieffermann [52] and was developed as a variant of free choice profiling (FCP). This sensory method combines the free generation of vocabulary through FCP and a ranking task with the simultaneous presentation of samples. Liu et al. [53] explained that FP provides both qualitative (generation of descriptors) and quantitative (intensity ranking task) information. The FP assessment process is similar to the FCP process and starts with the generation of sensory attributes that best describe samples. Panelists then evaluate the entire set of samples and rank them for each attribute generated according to their intensity.

Descriptors obtained from an FP are collected on a table in order to create a matrix where rows correspond to attributes and columns to wines with, at the intersection of the rows and columns, the number of citations for each descriptor. Data analysis is performed with GPA to evaluate consensus among participants. Ranking data are then analyzed using Friedman’s nonparametric test. Finally, a HCA is applied to the descriptors to perform the semantic interpretation.

Determining differences between rapid sensory techniques and conventional profiling (CP), Delarue and Sieffermann [54] assumed that FP was faster, while CP provided information on a larger number of descriptors. Liu et al. [53] compared the use of FP with a trained and an untrained panel on white wine models, concluding that the trained panel gave more accurate, robust and reliable results. After comparing FCP, FP, and CP applied to red wines from Merlot, Liu et al. [55] observed that the lists of attributes generated through each profiling technique were similar. FP showed greater discriminative ability compared to FCP and CP. However, panelists from the Departments of Food Science and Technology and Viticulture and Enology at UC Davis considered FCP to be easier to perform than FP. To conclude with the comparative studies, Delarue and Sieffrermann [56] suggested that FP could not replace conventional profiles, which they considered to be the best adapted and most accurate descriptive profiling methods.

#### 4.2.3. Ultra-Flash Profiling

Ultra-flash profiling (UFP) is generally not used alone, but after Napping, in order to briefly describe the sensory properties of different samples. The technique is the least time-consuming and provides an easy descriptive method for sample characterization. UFP appears to be the best adapted sensory method to complete Napping [57]. The UFP assessment process is very simple. Participants write terms directly on their tablecloth next to where the samples have been positioned in order to describe them. 

Data obtained after UFP are collected on a global table where rows correspond to samples and columns to attributes. Experimenters analyze the Napping tablecloth for each participant and for each wine and assign a “1” value if a descriptor is cited for the wine and a “0” value if not [57]. The UFP data are analyzed using GPA, as previously mentioned (cf. Section 4.2.1. Free Choice Profiling and Section 4.2.2. Flash Profile), to assess consensus between descriptors generated by the assessors and comparison of the proximity between the terms used by the different assessors to describe samples [58]. Finally, HCA is applied to gather groups of samples according to their similarity regarding sensory attributes generated by participants.

Perrin et al. [38] compared three profiling approaches (CP, FP, and UFP) to combine them with Napping in order to characterize the sensory space of red wines from the Central Loire Valley in France with a panel of wine professionals. The authors concluded that the UFP descriptive method was the most suitable to combine with Napping as it provided a rapid and easy way to profile wine samples directly on a tablecloth. CP was difficult to adapt to wine professionals as they were too busy to take part in efficient extensive training. FP was also considered too time-consuming as the assessors had to generate their own list of descriptors before rating their intensity. In addition, Perrin and Pagès [58] used a panel of UFP experts combined with Napping to describe the sensory space of red wines from the Central Loire Valley. This approach demonstrated that the data obtained by UFP were interpretable and rich in information.

### 4.3. Citation Frequency Method

Conventional profiling (CP) can be replaced by a variant based on attribute citation frequencies instead of attribute intensity rating. The aim is to identify the most relevant attributes chosen by participants according to their citation frequencies. Training for the citation frequency method is similar to the CP process. The number of times a descriptor is generated (called occurrence) and the percentage of tasters that cite each descriptor is determined. Attributes cited by more than 15% by the panel are then organized into a contingency table, after which a correspondence analysis is applied to transform this table into a graph and to analyze the attribute citation frequencies. Finally, HCA is performed to identify groups of samples formed in a similar way by the participants. 

Campo et al. [30] and Nanou et al. [59] used the citation frequency method to investigate the aroma properties of Spanish and Greek monovarietal white wines, respectively. Campo et al. [60] were the first to compare the CP and the citation frequency method. They observed that the main difference between the two was the average number of panelists required: to obtain significant and representative results from a citation frequency method, the number of participants should be higher (around 30) than for CP (where around 10 is sufficient).

Finally, many sensory methodologies can be adapted to the study of sensory spaces. In a recent study, for example, Leriche et al. [61] used just-about-right scales, a classic methodology used in consumer preference studies. The authors demonstrated that this method could be adapted to the typicity study, where wine professionals evaluate the intensity of descriptors in comparison with an ideal type.

**Table 2:** Inventory of perceptual methodologies used to describe sensory space. Statistical analysis—ANOVA: analysis of variance; PCA: principal component analysis; CA: correspondence analysis; GPA: generalized procrustes analysis; HCA: hierarchical cluster analysis.

Sensory Tests	Data Collected	Statistical Analysis	Advantages	Disadvantages
**Conventional profiling (CP) [43]**	Generation of descriptors and intensity rating	❖ ANOVA ❖ PCA	❖ This is the most reliable and accurate method. ❖ Involves a trained panel [44]. ❖ Provides consistent and reproducible results.	❖ Time-consuming because of the extensive training sessions. ❖ Evaluating a large number of samples is tiring.
**Free choice profiling (FCP) [49]**	Free generation of descriptors	❖ χ^2^ test ❖ CA ❖ GPA	❖ Is faster and more flexible than CP [50]. ❖ No specific training is required [50]. ❖ Is adapted to wine professionals. ❖ Takes into account the interindividual variation in perceptions.	❖ Is less accurate than CP [50]. ❖ The diversity of descriptors induces some difficulties in statistical treatment.
**Flash profiling (FP) [52]**	Free generation of descriptors and intensity ranking	❖ GPA ❖ Friedman’s nonparametric test ❖ HCA	❖ Is faster than CP [54]. ❖ No specific training is required [53]. ❖ Is adapted to wine professionals. ❖ Takes into account the interindividual variation in perceptions.	❖ Is less accurate than CP [54]. ❖ Is more difficult than FCP [55]. ❖ The diversity of descriptors induces some difficulties in statistical treatment.
**Ultra-flash profiling (UFP) [57]**	Free generation of descriptors	❖ GPA ❖ HCA	❖ Is the least time-consuming method [38]. ❖ Is an easy descriptive method. ❖ Is the most suitable method to combine with Napping [38]	❖ Used only in combination with Napping [57]. ❖ Less accurate than CP.
**Citation frequency method [30]**	Generation of descriptors and citation frequencies	❖ CA ❖ HCA	❖ Identifies the most relevant attributes. ❖ Involves a trained panel [44] (without training on the intensity scale).	❖ Involves a higher number of participants than CP (around 30 participants) [30]. ❖ Is time-consuming because of the training sessions. ❖ No indication regarding intensity of each attribute.

## 5. Conclusions

The inventory of conceptual and perceptual approaches involves three main methods that can be used to identify, define, and characterize wine sensory spaces with regard to typicality, ageing potential, minerality, complexity, woody character, etc.

Conceptual approaches combined with sensory methods, such as sorting tasks or Napping, can be used for this purpose. This type of sensory technique, based on similarity and dissimilarity assessment, is useful for discriminating products and proving the existence of sensory boundaries between them. The sensory space of a product is thus defined when sensory borders are well defined. However, the boundaries of this definition are taster-dependent. Sorting tasks and Napping provide a spatial representation of sensory products, potentially highlighting groups. Thus, samples that are close to each other are considered similar from a sensory point of view. The sorting task and Napping are generally associated with a verbalization task to explain the assessors’ sorting strategy. These sorting methods have the advantage of being very simple since they require no specific training and can be quickly performed by assessors. However, such sensory methods reveal an incomplete sensory description of samples, as participants do not use a wide and accurate list of descriptors. 

After determining the sensory boundaries of a sensory space, it is interesting to describe it using common perceptual approaches of descriptive analysis that include profiling methods such as conventional profiling, free choice profiling, or flash profiling. Conventional profiling provides a very accurate sensory characterization of samples. However, the main disadvantage of this method compared to the others is that it is time-consuming due to the use of extensive training to ensure that the terminology is used consistently. Other descriptive methods have been proposed that are better adapted to use by wine professional panels with global expertise in tasting.

Finally, the study of wine sensory spaces related to quality, identity, or typicality could have applications in the promotion of wines to consumers, and often leads to additional approaches dedicated to the characterization of compounds at the origin of specific sensory expressions.

## Data Availability

Data sharing not applicable.
